# Transgender Identity and Experiences of Violence Victimization, Substance Use, Suicide Risk, and Sexual Risk Behaviors Among High School Students — 19 States and Large Urban School Districts, 2017

**DOI:** 10.15585/mmwr.mm6803a3

**Published:** 2019-01-25

**Authors:** Michelle M. Johns, Richard Lowry, Jack Andrzejewski, Lisa C. Barrios, Zewditu Demissie, Timothy McManus, Catherine N. Rasberry, Leah Robin, J. Michael Underwood

**Affiliations:** ^1^Division of Adolescent and School Health, National Center for HIV/AIDS, Viral Hepatitis, STD, and TB Prevention, CDC; ^2^Oak Ridge Institute for Science and Education, Oak Ridge, Tennessee.

Transgender youths (those whose gender identity* does not align with their sex^†^) experience disparities in violence victimization, substance use, suicide risk, and sexual risk compared with their cisgender peers (those whose gender identity does align with their sex) (*1*–*3*). Yet few large-scale assessments of these disparities among high school students exist. The Youth Risk Behavior Survey (YRBS) is conducted biennially among local, state, and nationally representative samples of U.S. high school students in grades 9–12. In 2017, 10 states (Colorado, Delaware, Hawaii, Maine, Maryland, Massachusetts, Michigan, Rhode Island, Vermont, Wisconsin) and nine large urban school districts (Boston, Broward County, Cleveland, Detroit, District of Columbia, Los Angeles, New York City, San Diego, San Francisco) piloted a measure of transgender identity. Using pooled data from these 19 sites, the prevalence of transgender identity was assessed, and relationships between transgender identity and violence victimization, substance use, suicide risk, and sexual risk behaviors were evaluated using logistic regression. Compared with cisgender males and cisgender females, transgender students were more likely to report violence victimization, substance use, and suicide risk, and, although more likely to report some sexual risk behaviors, were also more likely to be tested for human immunodeficiency virus (HIV) infection. These findings indicate a need for intervention efforts to improve health outcomes among transgender youths.

In the 2017 YRBS cycle, states and local urban school districts could pilot a question about transgender identity (Box). This question was developed by CDC survey methodologists with input from external experts in transgender health to create a single-item measure to assess the prevalence of transgender identity among high school students. Ten states and nine large urban school districts piloted this question, and these data were pooled for this analysis (131,901 students). Data were weighted to be representative of public school students attending grades 9–12 in each jurisdiction. Survey procedures protected students’ privacy, participation was anonymous and voluntary, and local procedures were followed to review and approve the YRBS and obtain parental consent.

BOX Transgender pilot question — Youth Risk Behavior Surveys, 10 U.S. states* and nine large urban school districts,^†^ 2017

Some people describe themselves as transgender when their sex at birth does not match the way they think or feel about their gender. Are you transgender?

A. No, I am not transgender

B. Yes, I am transgender

C. I am not sure if I am transgender

D. I do not know what this question is asking

* Colorado, Delaware, Hawaii, Maine, Maryland, Massachusetts, Michigan, Rhode Island, Vermont, and Wisconsin.

^†^ Boston, Massachusetts; Broward County, Florida; Cleveland, Ohio; Detroit, Michigan; District of Columbia; Los Angeles, California; New York City, New York; San Diego, California; and San Francisco, California.

To produce prevalence estimates for transgender identity, respondents were categorized based on responses to the pilot question into the following four groups: 1) No, I am not transgender; 2) Yes, I am transgender; 3) I am not sure if I am transgender; and 4) I do not know what this question is asking. To examine behavioral comparisons, respondents were categorized based on responses to the pilot question and the question about sex (“What is your sex?”) into the following three groups: 1) cisgender males (male, not transgender); 2) cisgender females (female, not transgender); and 3) transgender students. Because it is unclear whether transgender students’ responses to the sex question reflected their sex or gender identity, this analysis could not further disaggregate transgender students. Students who responded that they were not sure if they were transgender or that they did not know what the question was asking were excluded from behavioral comparisons.

Victimization was assessed by students’ responses to the following items: in the past 12 months 1) threatened or injured with a weapon at school; 2) experienced sexual dating violence; 3) experienced physical dating violence; 4) bullied at school; 5) electronically bullied; 6) in the past 30 days, felt unsafe at or traveling to or from school; or 7) ever forced to have sexual intercourse. Information on lifetime use of cigarettes, alcohol, marijuana, cocaine, heroin, methamphetamines, ecstasy, or inhalants, and prescription opioid misuse was collected. Suicide risk was assessed by responses to questions about whether, in the past 12 months, the student felt sad or hopeless, considered attempting suicide, made a suicide plan, attempted suicide, or had a suicide attempt treated by a doctor or nurse. Sexual risk behaviors were assessed by students’ responses to questions about whether they had ever had sexual intercourse; had first sexual intercourse before age 13 years; had sexual intercourse with four or more persons during their life; had sexual intercourse during the past 3 months (currently sexually active); did not use a condom during last sexual intercourse; did not use any method to prevent pregnancy during last sexual intercourse; drank alcohol or used drugs before last sexual intercourse; and had never been tested for HIV infection.

To examine the prevalence of transgender identity, unadjusted prevalence estimates with 95% confidence intervals (CIs) were calculated using Taylor series linearization for prevalence. To test for differences in behavioral outcomes by gender identity, logistic regression models, controlling for race/ethnicity, grade, and site (school district versus state) produced adjusted prevalence ratios (APRs) with cisgender male students serving as referent group. Post-hoc linear contrast t-tests were used to assess additional between-group differences in outcome prevalence by gender identity. Differences were considered statistically significant if p<0.05 or 95% CIs did not include 1.0.

Across the 19 sites, 94.4% (range = 94.0%–94.8%) of students responded “No, I am not transgender”; 1.8% (range = 1.0%–3.3%) responded “Yes, I am transgender”; 1.6% (range = 0.9%–2.5%) responded “I am not sure if I am transgender”; and 2.1% (range = 1.5%–4.7%) responded “I do not know what this question is asking.” (Table 1)

**TABLE 1:** Unweighted number and weighted percentage of transgender item responses — 10 U.S. states* and nine large urban school districts,^†^ Youth Risk Behavior Survey, 2017

Site	Transgender question response							
	No, I am not transgender		Yes, I am transgender		I am not sure if I am transgender		I do not know what this question is asking	
	No.	% (95% CI)	No.	% (95% CI)	No.	% (95% CI)	No.	% (95% CI)
Selected states (pooled)	90,415	94.6 (94.1–95.1)	2,359	1.9 (1.6–2.1)	2,020	1.6 (1.4–1.9)	1,998	1.9 (1.7–2.2)
Large urban school districts (pooled)	28,388	93.9 (93.3–94.5)	486	1.6 (1.4–2.0)	499	1.6 (1.4–1.8)	908	2.9 (2.5–3.2)
State and school district data (pooled)	118,803	94.4 (94.0–94.8)	2,845	1.8 (1.6–2.0)	2,519	1.6 (1.4–1.8)	2,906	2.1 (1.9–2.4)

* Colorado, Delaware, Hawaii, Maine, Maryland, Massachusetts, Michigan, Rhode Island, Vermont, and Wisconsin.

^†^ Boston, Massachusetts; Broward County, Florida; Cleveland, Ohio; Detroit, Michigan; District of Columbia; Los Angeles, California; New York City, New York; San Diego, California; and San Francisco, California.

The reported prevalence of all experiences assessing violence victimization was higher among transgender students than among both cisgender males and cisgender females, including 23.8% reporting ever being forced to have sexual intercourse and 26.4% having experienced physical dating violence (Table 2). A higher percentage of transgender students also reported lifetime use of all substances except marijuana than did cisgender male and cisgender female students; marijuana use was more prevalent among transgender students than among cisgender male students only. A higher proportion of transgender students reported all suicide risk outcomes than did cisgender students.

**TABLE 2:** Unadjusted prevalence (%) and adjusted prevalence ratios (APRs) of violence victimization, substance use, suicide risk, and sexual risk among cisgender male, cisgender female, and transgender students — 10 U.S. states* and nine large urban school districts,^†^ Youth Risk Behavior Surveys, 2017

Health risk behaviors or experiences (no. of sites that asked question)	Cisgender students				Transgender students	
	Males		Females			
	% (95% CI)	APR (95% CI)	% (95% CI)	APR (95% CI)^§^	% (95% CI)	APR (95% CI)^§^
**Violence victimization**						
Felt unsafe at or traveling to/from school (17)^¶^	4.6 (4.0–5.2)	1.0 (ref)	7.1 (6.3–8.0)	1.56** (1.33–1.82)	26.9 (21.4–33.1)	5.44**^,††^ (4.09–7.23)
Threatened or injured with a weapon at school (17)^§§^	6.4 (5.8–7.0)	1.0 (ref)	4.1 (3.6–4.6)	0.63** (0.55–0.73)	23.8 (20.0–28.1)	3.39**^,††^ (2.69–4.27)
Ever forced to have sexual intercourse (18)^¶¶^	4.2 (3.6–4.9)	1.0 (ref)	10.5 (9.5–11.6)	2.55** (2.09–3.11)	23.8 (19.0–29.3)	5.45**^,††^ (4.11–7.21)
Experienced sexual dating violence (15)***	3.5 (3.0–4.2)	1.0 (ref)	12.0 (10.8–13.3)	3.51** (2.91–4.24)	22.9 (17.4–29.5)	6.42**^,††^ (4.62–8.91)
Experienced physical dating violence (19)^†††^	5.8 (5.1–6.5)	1.0 (ref)	8.7 (8.0–9.4)	1.50** (1.32–1.71)	26.4 (21.1–32.5)	4.15**^,††^ (3.13–5.48)
Bullied at school (18)^§§§^	14.7 (13.8–15.7)	1.0 (ref)	20.7 (19.6–21.8)	1.42** (1.31–1.53)	34.6 (29.8–39.8)	2.33**^,††^ (1.95–2.78)
Electronically bullied (19)^¶¶¶^	10.2 (9.5–10.9)	1.0 (ref)	19.3 (18.5–20.2)	1.90** (1.76–2.05)	29.6 (24.4–35.4)	2.90**^,††^ (2.40–3.49)
**Substance use**						
Cigarettes, lifetime use (12)	23.2 (21.0–25.6)	1.0 (ref)	22.0 (19.9–24.3)	0.94 (0.86–1.03)	32.9 (26.4–40.2)	1.34**^,††^ (1.07–1.69)
Alcohol, lifetime use (16)	53.3 (51.3–55.4)	1.0 (ref)	62.8 (60.9–64.6)	1.17** (1.14–1.21)	70.0 (63.7–75.6)	1.31**^,††^ (1.20–1.43)
Marijuana, lifetime use (14)	34.1 (31.9–36.4)	1.0 (ref)	38.0 (35.7–40.3)	1.10** (1.05–1.16)	43.8 (36.9–51.0)	1.26** (1.06–1.49)
Cocaine, lifetime use (17)	4.3 (3.5–5.3)	1.0 (ref)	2.6 (2.2–3.0)	0.60** (0.47–0.76)	27.2 (22.8–32.0)	5.99**^,††^ (4.54–7.92)
Heroin, lifetime use (16)	2.2 (1.9–2.7)	1.0 (ref)	0.7 (0.5–1.0)	0.31** (0.22–0.43)	26.1 (22.2–30.3)	10.23**^,††^ (8.01–13.1)
Methamphetamines, lifetime use (13)	2.3 (1.8–2.9)	1.0 (ref)	1.0 (0.7–1.3)	0.42** (0.28–0.64)	24.9 (20.9–29.3)	9.75**^,††^ (7.05–13.5)
Ecstasy, lifetime use (12)	3.6 (3.1–4.2)	1.0 (ref)	2.4 (2.1–2.8)	0.67** (0.54–0.83)	31.6 (26.8–36.8)	7.87**^,††^ (6.03–10.3)
Inhalants (11)****	6.0 (4.9–7.5)	1.0 (ref)	4.9 (4.2–5.8)	0.82 (0.66–1.02)	31.1 (24.2–38.9)	4.79**^,††^ (3.43–6.67)
Prescription opioid misuse (17)^††††^	11.5 (10.2–12.9)	1.0 (ref)	12.3 (11.1–13.7)	1.08 (0.96–1.22)	35.9 (31.3–40.7)	2.95**^,††^ (2.41–3.60)
**Suicide risk**						
Felt sad or hopeless (19)^§§§§^	20.7 (19.8–21.7)	1.0 (ref)	39.3 (38.0–40.7)	1.91** (1.81–2.00)	53.1 (47.7–58.4)	2.58**^,††^ (2.32–2.87)
Considered attempting suicide (18)^¶¶¶¶^	11.0 (10.2–11.9)	1.0 (ref)	20.3 (19.3–21.3)	1.85** (1.70–2.01)	43.9 (38.3–49.7)	3.95**^,††^ (3.39–4.60)
Made a suicide plan (16)*****	10.4 (9.4–11.4)	1.0 (ref)	16.0 (15.2–16.8)	1.56** (1.42–1.72)	39.3 (33.1–45.8)	3.72**^,††^ (3.10–4.46)
Attempted suicide (18)^†††††^	5.5 (4.9–6.1)	1.0 (ref)	9.1 (8.3–10.1)	1.70** (1.51–1.93)	34.6 (27.1–42.9)	6.30**^,††^ (4.81–8.24)
Had a suicide attempt treated by a doctor or nurse (14)^§§§§§^	2.1 (1.8–2.5)	1.0 (ref)	2.5 (2.0–3.1)	1.24 (0.92–1.67)	16.5 (10.9–24.3)	7.55**^,††^ (4.79–11.9)
**Sexual risk**						
Ever had sexual intercourse (17)	35.4 (33.4–37.3)	1.0 (ref)	33.2 (31.4–35.0)	0.93** (0.89–0.98)	43.1 (35.1–51.4)	1.21^††^ (0.98–1.50)
Had first sexual intercourse before age 13 years (19)	4.5 (3.8–5.2)	1.0 (ref)	1.5 (1.3–1.8)	0.34** (0.28–0.42)	14.9 (11.0–19.9)	3.17**^,††^ (2.16–4.66)
Had sexual intercourse with ≥4 persons (18)	8.9 (7.8–10.2)	1.0 (ref)	5.9 (5.2–6.7)	0.66** (0.56–0.78)	16.4 (11.9–22.0)	1.64**^,††^ (1.11–2.42)
Currently sexually active (18)^¶¶¶¶¶^	23.1 (21.4–24.8)	1.0 (ref)	25.8 (24.1–27.6)	1.11** (1.05–1.18)	27.8 (21.8–34.7)	1.21 (0.94–1.57)
Did not use condom during last sexual intercourse (18)	37.6 (34.9–40.5)	1.0 (ref)	48.9 (45.8–52.0)	1.30** (1.19–1.42)	63.8 (49.9–75.6)	1.69** (1.33–2.15)
Did not use any method to prevent pregnancy during last sexual intercourse (18)******	12.8 (10.8–15.0)	1.0 (ref)	13.0 (11.1–15.1)	1.06 (0.87–1.30)	29.7 (21.5–39.5)	2.20**^,††^ (1.50–3.23)
Drank alcohol or used drugs before last sexual intercourse (17)	19.2 (17.0–21.6)	1.0 (ref)	17.9 (15.9–20.1)	0.91 (0.76–1.09)	30.0 (20.8–41.2)	1.48^††^ (0.99–2.21)
Never been tested for HIV (16)^††††††^	87.4 (86.2–88.5)	1.0 (ref)	86.9 (85.8–87.9)	1.00 (0.98–1.01)	70.0 (64.4–75.0)	0.82**^,††^ (0.76–0.89)

**Abbreviations:** CI = confidence interval; HIV = human immunodeficiency virus; ref = referent.

* Colorado, Delaware, Hawaii, Maine, Maryland, Massachusetts, Michigan, Rhode Island, Vermont, and Wisconsin.

^†^ Boston, Massachusetts; Broward County, Florida; Cleveland, Ohio; Detroit, Michigan; District of Columbia; Los Angeles, California; New York City, New York; San Diego, California; and San Francisco, California.

^§^ Statistical significance is indicated when p<0.05 or 95% CI for APR does not include 1.0; % = unadjusted prevalence; APR adjusted for race/ethnicity, grade, and site. Referent group is cisgender male students. Each outcome was assessed by more than half of the 19 sites. Items “did not use a condom during last sexual intercourse,” “did not use any method to prevent pregnancy,” and “drank alcohol or used drugs before last sex” were tested only among sexually active students.

^¶^ Did not go to school because they felt unsafe at school or on their way to or from school on at least 1 day during the 30 days before the survey.

** Significantly different from cisgender male students.

^††^ Significantly different from cisgender female students.

^§§^ Threatened or injured with a weapon on school property during the 12 months before the survey.

^¶¶^ Ever physically forced to have sexual intercourse when they did not want to.

*** Being forced to do sexual things they did not want to do by someone they were dating or going out with ≥1 times during the 12 months before the survey, among students who dated or went out with someone during the 12 months before the survey.

^†††^ Being physically hurt on purpose by someone they were dating or going out with ≥1 times during the 12 months before the survey, among students who date or went out with someone during the 12 months before the survey.

^§§§^ Bullied on school property during the 12 months before the survey.

^¶¶¶^ Bullied through texting, Instagram, Facebook, or other social media during the 12 months before the survey.

**** Sniffed glue, breathed the contents of aerosol spray cans, or inhaled any paints or sprays to get high, one or more times during their life.

^††††^ Ever took prescription pain medicine without a doctor's prescription or differently than how a doctor told you to use it? (Counts drugs such as codeine, Vicodin, OxyContin, Hydrocodone, and Percocet.)

^§§§§^ Felt so sad or hopeless almost every day for ≥2 weeks in a row that they stopped doing some usual activities during the 12 months before the survey.

^¶¶¶¶^ Seriously considered attempting suicide during the 12 months before the survey.

***** Made a plan about how they would attempt suicide during the 12 months before the survey.

^†††††^ Attempted suicide one or more times during the 12 months before the survey.

^§§§§§^ Attempted suicide that resulted in an injury, poisoning, or overdose that had to be treated by a doctor or nurse during the 12 months before the survey.

^¶¶¶¶¶^ Had sexual intercourse with at least one person during the 3 months before the survey.

****** Question asked about method used for pregnancy prevention by “you or your partner.”

^††††††^ Never been tested for human HIV, the virus that causes acquired immunodeficiency syndrome (AIDS) (does not count if donated blood).

Transgender students were more likely than cisgender students to report first sexual intercourse before age 13 years, sexual intercourse with four or more persons than were cisgender students, and no method to prevent pregnancy at last sexual intercourse. Transgender students were more likely than were cisgender females to have ever had sex (43.1% versus 33.2%) and to have drunk alcohol or used drugs before their last sexual intercourse (30.0% versus 17.9%). Transgender students were more likely than were cisgender males to report no condom use during their last sexual intercourse (63.8% versus 37.6%). Transgender students were less likely than cisgender males and cisgender females to have not ever been tested for HIV (70.0% versus 87.4% and 86.9%, respectively).

## Discussion

Overall, 1.8% of students enrolled in the participating 10 state and nine urban school districts identified as transgender. This finding is consistent with previous studies of the prevalence of transgender identity among adolescents (*4*,*5*) and points to the utility of this measure to assess transgender identity broadly in a population-based study. Of note, some researchers recommend use of a sex question that includes a definition of sex as well as a gender identity question with five or more options (i.e., the two-step approach) to reliably characterize an individual’s current gender (*6*); such refined measures might benefit researchers in assessing within-group differences among transgender persons and aid in better targeting public health interventions.

The results of this study validate findings from smaller clinical and web-based studies that, at a population level, transgender students are at disproportionately higher risk than are cisgender students for violence victimization, substance use, and suicide risk (*1*–*3*). The prevalence of reported substance use (e.g., 27.1%, 26.1%, 24.9%, and 35.9% reporting lifetime use of cocaine, heroin, methamphetamines, and prescription opioid misuse, respectively) and suicide risk (e.g., 34.6% attempting suicide in the last 12 months) are concerning. Given that violence victimization is a documented risk factor for substance use and suicide risk (*7*), implementation of interventions focused on reducing the victimization of transgender adolescents might be a key strategy for improving overall health.^§^

Some examples of elevated sexual risk emerged among transgender students. More transgender than cisgender students reported first sexual intercourse before age 13 years and having had four or more sex partners, and more transgender students than cisgender female students reported ever having had sexual intercourse and use of alcohol or drugs before last sexual intercourse. Transgender students were more likely than were cisgender students to forego pregnancy prevention at last sexual intercourse and were less likely than were cisgender males to use a condom at last sexual intercourse; however, without further information about the sex and gender identities of these youths and their partners, the risk implications of these results are uncertain and should be interpreted with caution. Transgender students were more likely to have ever received an HIV test, an important protective behavior, given the known higher HIV risk experienced by this population (*3*).

The findings in this report are subject to at least three limitations. First, because of uncertainty as to whether transgender students responded to the sex question with their sex or gender identity, this analysis could not disaggregate transgender students to explore within-group differences in behavioral outcomes (e.g., a transgender student who was assigned the sex male at birth but currently identified as female might not know what response to provide on the existing sex question). Second, because YRBS is a school-based survey, students with the highest risk for these outcomes might have dropped out, and analyses might underestimate observed associations between risk behaviors and transgender identity (*8*). Finally, because YRBS is a cross-sectional survey, causation cannot be inferred from the findings.

Transgender youths in high school appear to face serious risk for violence victimization, substance use, and suicide, as well as some sexual risk behaviors, indicating a need for programmatic efforts to better support the overall health of transgender youths. Taking steps to create safe learning environments (*9*) and provide access to culturally competent physical and mental health care (*10*) might be important first steps to improving the health of transgender youths. Continued research into the health of transgender youths and development of effective intervention strategies are warranted.

**Summary**

**What is already known about this topic?**

Convenience samples indicate that transgender youths appear to be at higher risk for violence victimization, substance use, suicide risk, and sexual risk behaviors than are cisgender youth.

**What is added by this report?**

Population-based survey data from 10 state and nine urban school districts found that an average of 1.8% of high school students identify as transgender. Transgender students were more likely than were cisgender students to report violence victimization, substance use, and suicide risk, and, although generally more likely to report sexual risk behaviors, were also more likely to report having been tested for human immunodeficiency virus.

**What are the implications for public health practice?**

Coordinated intervention efforts to improve health outcomes among transgender youth are warranted.

## Footnotes

* Gender identity refers to an individual’s sense of their self as male, female, transgender, or something else. Gender identity is distinct from, but related to, gender, or the cultural roles, behaviors, activities, and attributes expected of women and men based on their sex.

† Sex refers to an individual’s biologic status as male, female, or something else. Sex is assigned to individuals at birth, and is associated with physical attributes, such as anatomy and chromosomes. This definition was not offered in the YRBS questionnaire.

§ CDC has a suite of violence prevention technical packages which can be adapted for such use. https://www.cdc.gov/violenceprevention/pub/technical-packages.html.

## References

[1] Reisner SL, Greytak EA, Parsons JT, Ybarra ML. Gender minority social stress in adolescence: disparities in adolescent bullying and substance use by gender identity. J Sex Res 2015;52:243–56. 10.1080/00224499.2014.886321 24742006 PMC4201643

[2] Reisner SL, Vetters R, Leclerc M, Mental health of transgender youth in care at an adolescent urban community health center: a matched retrospective cohort study. J Adolesc Health 2015;56:274–9. 10.1016/j.jadohealth.2014.10.264 25577670 PMC4339405

[3] Stieglitz KA. Development, risk, and resilience of transgender youth. J Assoc Nurses AIDS Care 2010;21:192–206. 10.1016/j.jana.2009.08.004 20347346

[4] Dane County Youth Commission. Dane County youth assessment overview report. Madison, WI: Dane County Youth Commission; 2015. https://danecountyhumanservices.org/yth/dox/asmt_survey/2015/2015_exec_sum.pdf

[5] Almeida J, Johnson RM, Corliss HL, Molnar BE, Azrael D. Emotional distress among LGBT youth: the influence of perceived discrimination based on sexual orientation. J Youth Adolesc 2009;38:1001–14. 10.1007/s10964-009-9397-9 19636742 PMC3707280

[6] Gender Identity in U.S. Surveillance. Gender-related measures overview. Los Angeles, CA: The Williams Institute; 2013. https://williamsinstitute.law.ucla.edu/wp-content/uploads/GenIUSS-Gender-related-Question-Overview.pdf

[7] Meyer IH, Frost DM. Minority stress and the health of sexual minorities. In: Patterson CJ, D'Augelli AR, eds. Handbook of psychology and sexual orientation. New York, NY: Oxford University Press; 2013:252–66.

[8] Burton CM, Marshal MP, Chisolm DJ. School absenteeism and mental health among sexual minority youth and heterosexual youth. J Sch Psychol 2014;52:37–47. 10.1016/j.jsp.2013.12.001 24495493 PMC4058829

[9] Orr A, Baum J, Brown J, Gill E, Kahn E, Salem A. Schools in transition: a guide for supporting transgender students in K–12 schools. New York, NY: American Civil Liberties Union; San Leandro, CA: Gender Spectrum; Washington, DC: Human Rights Campaign Foundation; San Francisco, CA: National Center for Lesbian Rights; Washington, DC: National Education Association; 2015. https://www.genderspectrum.org/staging/wp-content/uploads/2015/08/Schools-in-Transition-2015.pdf

[10] National LGBT Health Education Center. Transgender health. Boston, MA: National LGBT Health Education Center, The Fenway Institute; 2018. https://www.lgbthealtheducation.org/topic/transgender-health/

